# Tangeretin Improves the Memory of *Swiss* Mice, Suggesting Potential Molecular Interventions Through Animal Behavior Assessments and In Silico Studies

**DOI:** 10.1002/brb3.70516

**Published:** 2025-05-05

**Authors:** Md. Sakib Al Hasan, Mohd Shahnawaz Khan, Arusha Ayub, Raihan Chowdhury, Emon Mia, Md. Shadin, Md. Showkot Akbor, Muhammad Torequl Islam, Md. Shimul Bhuia

**Affiliations:** ^1^ Department of Pharmacy Goapalganj Science and Technology University Gopalganj Bangladesh; ^2^ Bioinformatics and Drug Innovation Laboratory BioLuster Research Center Ltd. Gopalganj Bangladesh; ^3^ Department of Biochemistry King Saud University Riyadh Saudi Arabia; ^4^ Department of Medicine, College of Health Sciences University of Georgia Georgia USA; ^5^ Pharmacy Discipline Khulna University Khulna Bangladesh

**Keywords:** dopamine receptor, memory‐enhancing effect, molecular interactions, tangeretin

## Abstract

**Introduction:**

Tangeretin (TAN), a polymethoxylated flavone from citrus peels, exhibits neuroprotective, anti‐inflammatory, and antioxidant properties. This study aims to evaluate the memory‐enhancing effects of TAN in *Swiss* mice and explore its potential molecular interactions with the D_2_ dopamine (DOP) receptor through in vivo behavioral assessments and in silico approaches.

**Methods:**

*Swiss* mice were administered TAN (10 and 20 mg/kg), DOP (22 mg/kg), and olanzapine (OLN) (2 mg/kg), alone and in combinations per orally (p.o.), followed by cognitive assessments using marble burying, dust removal, and trained swimming tests. In silico studies included molecular docking against the D_2_ receptor (PDB: 6CM4), pharmacokinetics (SwissADME, pkCSM), and toxicity predictions (ProTox‐3).

**Results:**

TAN significantly (*p* < 0.05) improved cognitive functions, including memory, anxiety, and motor coordination, in a dose‐dependent manner, with 20 mg/kg showing the most notable effect. The combination of TAN‐10 with DOP‐22 enhanced these benefits, whereas TAN‐10 with OLN‐2 reduced cognitive improvements. TAN‐treated Swiss mice showed better performance in marble burying, dust removal, and trained swimming tests, indicating enhanced memory, problem‐solving, and motor coordination. These results suggest TAN's potential in cognitive enhancement, particularly with DOP‐22. No deaths were observed in any treatment group, and all treated animals exhibited normal physiological activity with no signs of acute toxicity. In silico studies revealed that TAN exhibited the strongest binding affinity (BA) (−6.6 kcal/mol) with the D_2_ receptor, forming multiple hydrogen bonds (HBs), which indicates its potential mechanism for memory enhancement via dopaminergic modulation. Pharmacokinetic analyses also showed that TAN has favorable ADMET properties, including high gastrointestinal absorption, blood–brain barrier penetration, and low toxicity.

**Conclusion:**

These findings highlight TAN's potential as a promising therapeutic candidate for memory‐related disorders, warranting further clinical exploration.

## Graphical abstract

Swiss mice treated with tangeretin showed better performance in marble burying, dust removal, and trained swimming tests, resulting elevation of memory performance, problem‐solving, and motor coordination. The compound also demonstrated a remarkable binding affinity toward the D2 receptor with favorable pharmacokinetic properties in in silico analysis indicating as protential therapeutic agent.

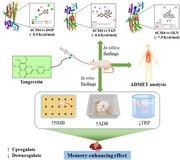

**Abbreviations**
ADMET: absorption, distribution, metabolism, excretion, and toxicity
AMES: *Salmonella typhimurium* reverse mutation assay
ANOVA: analysis of variance
AZD: Alzheimer's disease
BA: binding affinity
BBB: blood–brain barrier
BZDs: benzodiazepines
CNS: central nervous system
CYP P450: cytochrome P450
DOP: dopamine
HB: hydrogen bond
HIA: human intestinal absorption
HPLC: high‐performance liquid chromatography
LD_50_
: median lethal dose
OLN: olanzapine
PDB: protein data bank
SD: standard deviation
TAN: tangeretin

## 1 Introduction

Neurodegenerative disorders are defined by a progressive decrease in cognitive function that affects learning and memory failure in daily tasks (Blennow et al. 2006). Memory impairment may arise from neurological diseases such as Alzheimer's disease (AZD) (Baral et al. 2015) and Parkinson's disease (PD) (Ross and Poirier 2004), in addition to aging and stress. Amyloid plaque buildup, tau protein aggregation, cerebral oxidative stress, neuroinflammation, and cholinergic dysfunction are the causes of neurodegenerative diseases, including AZD. These conditions are accompanied by psychological and pathophysiological complications like anxiety, depression, difficulty concentrating, and motor disturbances (Lee et al. 2015). The risk of cognitive impairment is significantly increased with age. In Finland, moderate to severe dementia affects around 5% of those 65–75 years old, 10% of people 74–84 years old, and almost a third of people over 85 years old (Halonen et al. 2023). There was a 5.1%–41% worldwide prevalence of cognitive impairment, with a 19.0% median (Pais et al. 2020). Brain damage, mental disease, and neurological illnesses are examples of environmental variables that can induce cognitive deficiencies, which can also occur from birth (Mendola et al. 2002). Some of the early causes of cognitive deficit include chromosome abnormalities/genetic syndromes, prenatal drug exposure, malnutrition, poisoning due to lead or other heavy metals, neonatal jaundice, hypoglycemia, hypothyroidism, prematurity, hypoxia, trauma, or child abuse (Dhakal and Bobrin 2023).

DOP pathways were identified as being crucial to cognition and emotion in the field of catecholamine transmitters (Jay 2003), which directly affects synaptic memory formation and is recognized to be important for motivated behavior (Shohamy and Adcock 2010). Medication that may interfere with neurotransmitter activity (cholinergic, dopaminergic, noradrenergic, serotonergic, or GABAergic transmission) is likely to affect aspects of learning and memory (Garofalo et al. 1996).

There are a few synthetic medicines, for example, donepezil, galantamine, rivastigmine, and memantine, for treatment of cognitive dysfunction and memory loss. In addition, these compounds have been reported to have adverse effects, including nausea (Seltzer 2005), vomiting (Pratt et al. 2002), loss of appetite (Mimica and Presečki 2009), headache (Onor et al. 2007), constipation (Hoffman and Bloemer 2021), confusion (Da Re et al. 2015), and dizziness (Kavirajan 2009). Therefore, there is a need for an alternative treatment that is cheaper, more efficient, and has fewer side effects.

Natural products are important due to their diverse bioactive compounds, potential for fewer side effects, and historical use in traditional medicine (Aktar et al. 2024; Bithi et al. 2025). They serve as a crucial source for drug discovery, offering holistic and effective therapeutic options (Rakib et al. 2025; Sharma et al. 2025). In this regard, plants are the main resource because they may improve mood, sleep, and cognitive difficulties as well as enhance everyday activities (Sau and Handral 2015). Medicinal plants that can be used to treat memory impairment include *Hypericum perforatum*, *Lepidium meyenii*, *Cyperus rotundus*, *Zizyphus jujube*, *Lavandula officinalis*, *Ginkgo biloba*, *Salvia officinalis*, *Melissa officinalis*, *ginseng*, *Morinda citrifolia*, *Lycopodium serratum*, *Polygala tenuifolia*, and *Celastrus paniculatus* (Jivad and Rabiei 2014).

Tangeretin (TAN: 4′,5,6,7,8‐pentamethoxyflavone) is extensively found in citrus peels (Barreca et al. 2020). It has many important biological activities, including antioxidant (Wang et al. 2018), anxiolytic (Husain et al. 2024), sedative (Al Hasan, Bhuia, Chowdhury et al. 2025), anti‐inflammatory and antiasthmatic (Yumnam et al. 2018), neuroprotective (Yang et al. 2017), and anticancer (Raza et al. 2020) potentials. However, the memory‐enhancing capacity of TAN was not explored. Dopamine (DOP) modulation is crucial for cognitive function, and DOP is included to assess if enhancing dopaminergic signaling improves memory. In contrast, olanzapine (OLN) is used as a DOP D_2_ receptor antagonist to inhibit dopaminergic signaling, helping to study the opposing effects of reduced DOP activity on cognitive functions like memory (Islam, Chowdhury et al. 2024). TAN's interaction with the D_2_ receptor is explored as a potential memory enhancement mechanism, and comparing its effects with DOP and OLN will clarify if TAN acts as a DOP modulator.

This study aimed to evaluate the memory‐enhancing effect of TAN and explore its potential molecular mechanism through interactions with the DOP receptor.

## 2 Materials and Methods

### 2.1 In Silico Studies

#### 2.1.1 Receptor Selection and Preparation

The three‐dimensional structure of the selected macromolecule (D_2_ receptor) was obtained from the RCSB Protein Data Bank (https://www.rcsb.org/; retrieved on September 22, 2024). The macromolecules were refined by removing water molecules and extraneous amino acid residues using PyMOL version 1.7.4.5 (Bhuia et al. 2023). Minimized the energy of the crystal structure before docking by using the Swiss‐PDB Viewer software program (version 4.1.0, Swiss Institute of Bioinformatics, Biozentrum, Basel) (Jahan et al. 2025).

#### 2.1.2 Ligand Preparation

**FIGURE 1:**
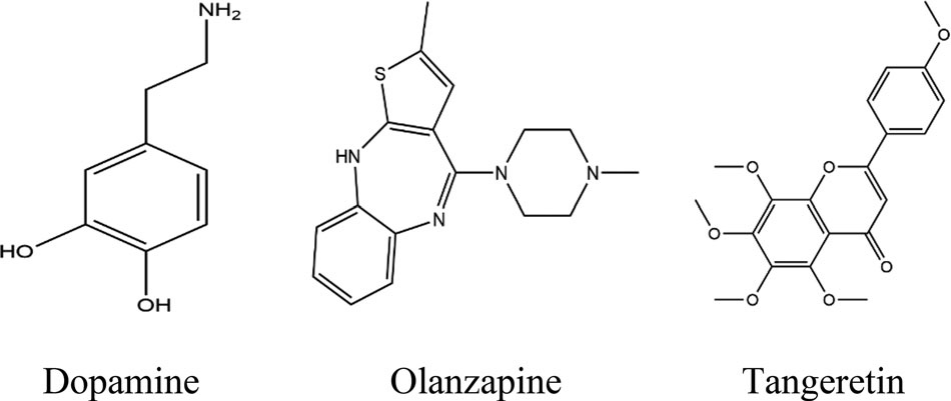
The 2‐dimensional chemical structures of tangeretin, dopamine, and olanzapine.

The standard medicine DOP (PubChem ID: 681), OLN (PubChem ID: 135398745), and TAN (PubChem ID: 68077) were obtained from the PubChem (https://pubchem.ncbi.nlm.nih.gov/; accessed on September 22, 2024) chemical database in the format of SDF files. The Chem3D 16.0 application package was then used to perform molecular docking on the 3D conformers of the chemical agents, minimizing them and saving them as SDF files (Chowdhury et al. 2024). The two‐dimensional images of the chemical agents are displayed in Figure 1.

#### 2.1.3 Docking Protocol and Non‐Bond Interactions

Molecular docking was done using the PyRx software program to determine the energy needed by a ligand to engage with the active sites of its receptor (Islam, Bappi et al. 2024). The *x*‐, *y*‐, and *z*‐axes’ respective grid box dimensions were determined to be 22.35 × 28.42 × 25.00 Å to ensure efficient docking. Two thousand steps were taken in the docking computation (Ibrahim et al. 2022). The receptor–ligand complex is put together in PDB format. The docking affinity result was recorded in a comma‐separated values (CSV) file. Additionally, the ligand is collected in PDBQT format for further analysis (Pawar and Rohane 2021). PyMOL (v2.5.8) and Discovery Studio Visualizer (v21.1.020298) were used to depict the interactions that occur between ligands and the receptor's active site (Hasan et al. 2024; Ghosh et al. 2025). Subsequently, all of the bond kinds, total quantity and duration of hydrogen bonds (HBs), and residues of amino acids involved in each interaction of a ligand with a receptor are recorded.

#### 2.1.4 Prediction of Drug‐Likeness, Pharmacokinetics, and Toxicity

Drug discovery relies heavily on the in silico assessment of ADMET qualities because it ensures an assessment of likely pharmacokinetic features and improved bioavailability (Al Hasan et al. 2024). The SwissADME (Akbor et al. 2023) and pkCSM (Islam et al. 2025) online platforms were used to investigate the physicochemical properties of potential candidates, including water solubility, lipid affinity, and pharmacokinetics. The potential toxicological characteristics of TAN, DOP, and OLA may be predicted using the Protox‐3 web servers. The Protox‐3 web server was primarily used to assess the parameters of hepatotoxicity, carcinogenicity, immunotoxicity, mutagenicity, and cytotoxicity (Ferdous et al. 2024). The canonical SMILES were uploaded into the pkCSM, SwissADME, and ProTox‐3 servers, which were gathered from PubChem to assess the physiochemical, pharmacokinetic, and toxicological characteristics.

### 2.2 In Vivo Findings

#### 2.2.1 Chemicals and Reagents

TAN was purchased from Chengdu Alfa Biotechnology Co. Ltd. (China) (CAS: 481‐53‐8, purity: 98% HPLC), whereas DOP and OLN were kindly supplied by Drug International Ltd. and Square Pharmaceuticals PLC, Bangladesh. Tween 80 and NaCl required for this study were purchased from Merck (India).

#### 2.2.2 Experimental Animals

Adult male *Mus musculus* (*Swiss* mice; avg. b.w. 26–30 g, age: 6–8 weeks) gathered from the Animal House of Jahangirnagar University, Bangladesh, were randomly distributed into different groups. Before that, the animals were housed in an optimal environment (temperature: 26°C ± 2°C, relative humidity: 65%) for 7 days. They had free access to standard foods and water ad libitum. Studies were performed between 9:00 a.m. and 3:00 p.m. Animals involved in this experiment were fasted overnight. However, they were allowed free access to water only. This study was approved by the Animal Ethics Committee of Khulna University (KUAEC‐2023‐05‐09).

#### 2.2.3 Dose Selection

The test doses for this study of TAN (10 and 20 mg/kg, p.o.) were chosen from the previously existing literature (Yang et al. 2017). DOP and OLN were administered at 22 and 2 mg/kg (p.o.), respectively. The control group received the vehicle, consisting of distilled water containing 0.9% NaCl and 0.5% Tween 80, at a volume of 10 mL/kg. Before treatment, all animals were fasted overnight. The study design section (Table 1) shows the animal grouping and treatment protocol.

**TABLE 1:** Treatment groups with their details at 10 mL/kg volume of administration via intraperitoneal route.

Treatment groups		Description	Administration design
*Individual groups*	Control	Vehicle: distilled water containing 0.9% NaCl and 0.5% Tween 80	At a time
	DOP‐22	D‐dopamine (dopamine receptor agonist reference drug) at 22 mg/kg	At a time
	OLN‐2	Olanzapine (dopamine receptor antagonist reference drug) at 2 mg/kg	At a time
	TAN‐10	Tangeretin (test sample) at 10 mg/kg	At a time
	TAN‐20	Tangeretin (test sample) at 20 mg/kg	At a time
*Combination groups*	DOP‐22 + OLN‐2	D‐dopamine 22 mg/kg + olanzapine 2 mg/kg	One followed by another
	TAN‐10 + DOP‐22	Tangeretin 10 mg/kg + D‐dopamine 22 mg/kg	One followed by another
	TAN‐10 + OLN‐2	Tangeretin 10 mg/kg + olanzapine 2 mg/kg	One followed by another

*Note*: Control: vehicle (distilled water containing 0.9% NaCl and 0.5% Tween 80); (*n* = 5).

Abbreviations: DOP, dopamine; OLN, olanzapine; TAN, tangeretin.

#### 2.2.4 Memory‐Enhancing Effect Study in Mice

In this study, a total of 40 adult male *Swiss* mice were randomly divided into 8 groups, each containing 5 animals (*n* = 5). All behavioral experiments were conducted by a blinded observer to ensure unbiased data collection and analysis. Treatment groups and their details have been shown in Table 1.

#### 2.2.5 Marble‐Burying Study

This test was performed using the model developed earlier by Njung'e and Handley (1991), with some modifications (Njung'e and Handley 1991). Briefly, respective treatments were given to the animal groups. Then, the animals were individually placed in a plastic box (21 × 38 × 14 cm^3^), which contained 5‐cm‐thick sawdust bedding and 30 small glass marbles (diameter: 11–14 mm) arranged on the bedding, maintaining equal distance in four rows. Each animal was exposed for 5 min on the bed, and the number of buried marbles (NMB) was counted. Marble covered at least two‐thirds of its size by the animal's sawdust was considered “buried.” The NMB was recorded and expressed as mean ± standard deviation (SD).

#### 2.2.6 Dust Removal Study

In this case, in a plastic box (21 × 38 × 14 cm^3^), a 1‐L‐capacity plastic beaker containing a mixture of dust (sand and straw) was placed. It was tilted at one end with dirt so that the animal could easily remove dust from it. The beaker, along with its dust content, was pre‐weighed so that it could easily determine the removed weight. Thirty minutes after treatment with the respective substance, each animal was allowed to remove dust from the tilted beaker for 5 min. An animal's amount of dust removal (ADR) is measured in grams. The animals’ stools were removed from the dust after each performance. The ADR was measured in grams and presented as mean ± SD.

#### 2.2.7 Trained Swimming Study

This study used a modified method of Porsolt et al. (1977). In this case, mice were individually trained to swim in an open cylindrical container (diameter × height: 10 × 25 cm^2^) for 2 min. One side of the container contains a stainless steel (SS) rack, which is considered a target point so that the animal can hold it and escape from the water. The SS rack was placed just above the water top of the container. The container contains 19 cm of water at 25°C ± 1°C. Then, the animals were treated with respective agents (test or controls), and after 30 min, each animal was allowed to swim in the water. From the time of putting water, the time to reach the target point (TRP) was counted for each animal. The body of each animal was wiped with a dry cloth after each performance in water, and they were placed in a warm place. The TRP was recorded in seconds and expressed as mean ± SD.

#### 2.2.8 Statistical Analysis

Values are expressed as the mean ± SD. One‐way ANOVA followed by *t*‐students Newman–Keuls as a post hoc test with multiple comparisons at 95% confidence intervals using GraphPad Prism software (version: 9.5, San Diego, USA). Data were considered significant when *p* *< *0.05.

## 3 Results

### 3.1 In Silico Findings

#### 3.1.1 Molecular Docking and Visualization of Ligand–Receptor Interaction

In this study, the test compound TAN exhibited a binding affinity (BA) of −6.6 kcal/mol with the D_2_ receptor. It formed one HB with ALA A: 379 (2.75 Å) as an H‐acceptor and engaged in hydrophobic interactions with ALA A: 376 (4.18 Å), ILE A: 383 (4.67 Å), PHE A: 202 (5.47 Å), TYR A: 213 (4.11 Å), and LEU A: 206 (5.08 Å). The standard compound DOP showed a BA of −5.0 kcal/mol, forming three HBs with ASN A: 430 (2.80 Å, H‐donor) and additional HBs with ASN A: 70 (2.41 Å, H‐acceptor) and GLU A: 368 (2.14 Å, H‐acceptor). It also exhibited hydrophobic interactions with LYS A: 370 (4.18 Å) and ALA A: 371 (4.14 Å). Among the tested ligands, OLN exhibited the highest BA of −7.5 kcal/mol. It formed one HB with TYR A: 209 (2.57 Å, H‐acceptor) and showed hydrophobic interactions with ALA A: 376 (4.05 Å), ALA A: 379 (4.91 Å), ILE A: 210 (5.07 Å), and TYR A: 213 (4.19 Å).

**FIGURE 2:**
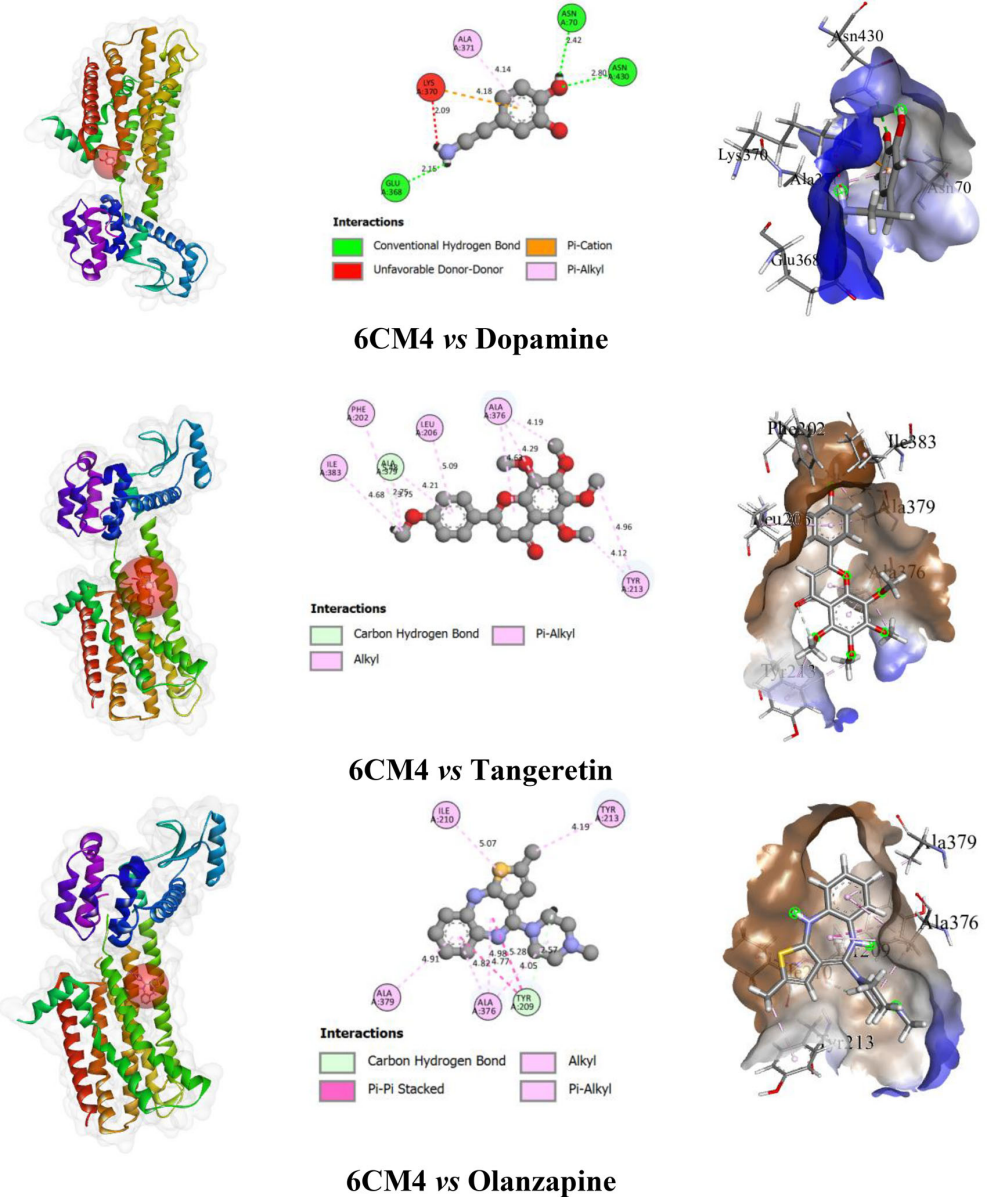
The 2D and 3D structures of the non‐bond interactions among dopamine, olanzapine, and tangeretin with the selected receptor (6CM4).

However, details of the BA, number of HBs and AA residues related to HB, and other types of bonds of ligands with the selected receptor are presented in Table 2. The binding pockets of 2D and 3D structures, including the interacting AA residues and bond types of ligands with the selected receptor, are depicted in Figure 2.

**TABLE 2:** The binding affinity and several types of bonds among tangeretin, dopamine, and olanzapine with D_2_ dopamine receptor.

Receptor (PDB ID)	Ligand	BA	No. of HB	Types of bonds	AA residues	Length of HB (Ǻ)	Role
D_2_ dopamine (6CM4)	TAN	−6.6	1	HB	ALA A: 379	2.75	H‐acceptor
				HPB	ALA A: 376	4.18	Alkyl
					ILE A: 383	4.67	Alkyl
					PHE A: 202	5.47	Pi‐orbitals
					TYR A: 213	4.11	Pi‐orbitals
					LEU A: 206	5.08	Alkyl
	DOP	−5.0	3	HB	ASN A: 430	2.80	H‐donor
					ASN A: 70	2.41	H‐acceptor
					GLU A: 368	2.14	H‐acceptor
				HPB	LYS A: 370	4.18	Positive
					ALA A: 371	4.14	Alkyl
	OLN	−7.5	1	HB	TYR A: 209	2.57	H‐acceptor
				HPB	ALA A: 376	4.05	Alkyl
					ALA A: 379	4.91	Alkyl
					ILE A: 210	5.07	Alkyl
					TYR A: 213	4.19	Pi‐orbitals

Abbreviations: AA, amino acid; DOP, dopamine; HB, hydrogen bond; HPB, hydrophobic bond; OLN, olanzapine; TAN, tangeretin.

#### 3.1.2 Pharmacokinetic and Drug‐Likeness and Toxicity Properties

On the basis of in silico ADMET, every ligand has an MW of less than 500 g/mol. According to Lipinski's rule of five, the molar refractivity (MR ≤ 140) and the HBD (≤5) and HBA (≤10) values are within the range. A bioavailability score of 0.55 is assigned to them. They both dissolve when submerged in water. The hydrophobic properties and lipid bilayer penetration of TAN, DOP, and OLN were shown by their respective log *p* values of 0.63, 0.60, and 2.88. All of these results (MLOGP3 < 4.15) are within the acceptable ranges. All of the ligands are highly absorbable across the GI membrane. All ligands can penetrate the blood–brain barrier and affect the central nervous system, except DOM. Furthermore, among the measures that correspond with the appropriate pharmacokinetic features are Pgp substrate status, CYP3A4 and CYP2D6 enzyme inhibition, log Kp, bioavailability score, plasma protein binding (PPB), drug clearance (CL), and half‐life (T1/2).

The Protox‐3 web server may be used to determine potential toxicity markers for medications. In silico toxicity predictions were used to determine the LD50 values for TAN (5000 mg/kg), OLA (200 mg/kg), and DOP (24,599 mg/kg). Furthermore, these findings determined that TAN, OLN, and DOM are classified as belonging to toxicity classes 5, 3, and 5. TAN did not show any toxicity on most of the organ toxicity predictions, except for nephrotoxicity, respiratory toxicity, BBB barrier, ecotoxicity, and nutritional toxicity. Among these were parameters related to carcinogenicity, cytotoxicity, immunotoxicity, hepatotoxicity, mutagenicity, respiratory toxicity, and mutagenicity. Conversely, DOP may have neurotoxicity, clinical toxicity, and cytotoxic effects, whereas CAR may cause neurotoxicity, respiratory toxicity, clinical toxicity, BBB‐barrier, and ecotoxicity.

**FIGURE 3:**
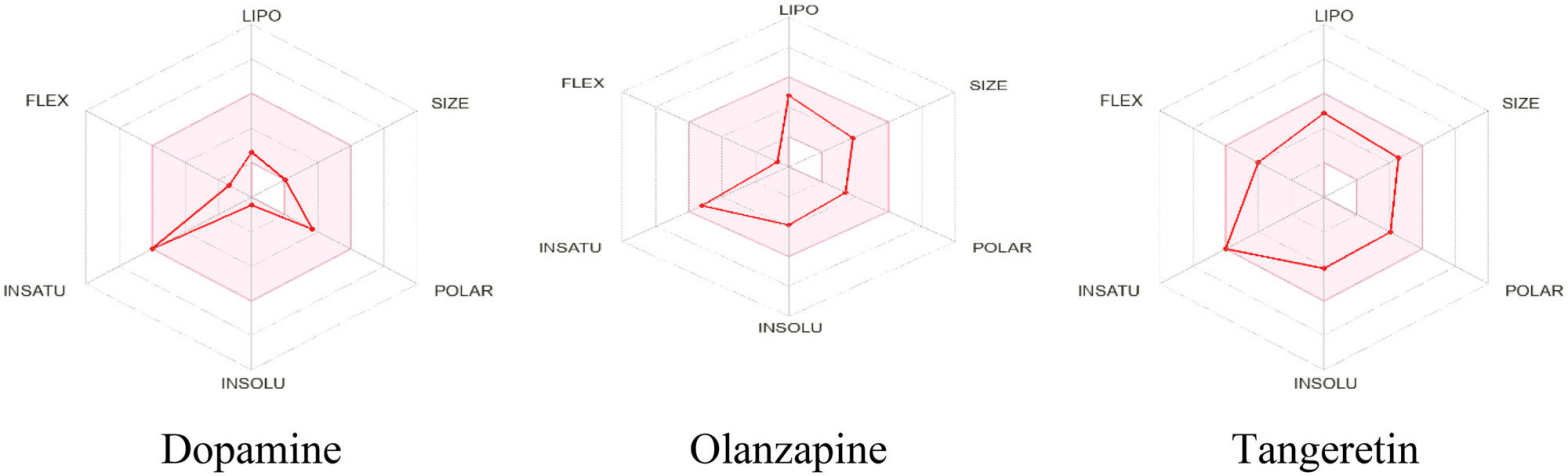
Bioavailability radar related to physicochemical properties of tangeretin, dopamine, and olanzapine [the physicochemical space that is appropriate for oral bioavailability is the colored zone; SIZE: 150 g/mol < MV < 500 g/mol; LIPO (lipophilicity): −7 < XLOGP3 < + 5.0; INSOLU (insolubility): −6 < log S (ESOL) < 0; POLAR (polarity): 20 Å^2^ < TPSA < 130 Å^2^; IN‐SATU (in saturation): 0.25 < fraction Csp3 < 1; FLEX (flexibility): 0 < num. rotatable bonds < 9].

In addition, Table 3 lists the pharmacokinetic and drug‐likeness properties of DOP, OLN, and TAN. Figure 3 shows a graphical representation of the physiochemical and pharmacokinetic properties of particular compounds.

**TABLE 3:** Pharmacokinetic and drug‐likeness properties of dopamine, olanzapine, and tangeretin.

Properties	Factors	Dopamine	Olanzapine	Tangeretin
Physicochemical properties	Formula	C_8_H_11_NO_2_	C_17_H_20_N_4_S	C_20_H_20_O_7_
	Molar mass (g/mol)	153.18 g/mol	312.43 g/mol	372.37
	Number of heavy atoms	11	22	27
	Number of aromatic heavy atoms	6	11	16
	Number of H‐bond donors	3	1	0
	Number of H‐bond acceptors	3	2	7
	Molar refractivity	42.97	107.89	100.38
Lipophilicity	Log Po/w (MLOGP)	0.60	2.88	0.63
Drug‐likeness	Lipinski	Yes; 0 violation	Yes; 0 violation	Yes; 0 violation
	Bioavailability score	0.55	0.55	0.55
Water Solubility	Log S (ESOL)	−0.44	−3.88	−4.11
	Class	Very soluble	Soluble	Moderately soluble
Absorption	Caco2 permeability (log Papp in 10–6 cm/s)	0.716	1.491	1.245
	Intestinal absorption (human) numeric (%absorbed)	75.815	91.841	98.478
	Skin permeability (log Kp cm/h)	−2.863	−2.737	−2.678
	P‐glycoprotein I inhibitor	No	No	Yes
	P‐glycoprotein II inhibitor	No	Yes	Yes
Distribution	BBB permeability (log BB)	−0.414	0.99	−1.026
	CNS permeability (log PS)	−2.65	−1.694	−3.011
	VDss (human) (log L/kg)	0.622	1.816	−0.226
Metabolism	CYP2D6 substrate	No	Yes	Yes
	CYP3A4 substrate	No	Yes	Yes
	CYP2D6 inhibitor	No	Yes	No
	CYP3A4 inhibitor	No	No	Yes
Excretion	Total clearance (log mL/min/kg)	0.842	0.659	0.78
	Renal OCT2 substrate	No	Yes	No
Toxicity	Hepatotoxicity	Inactive	Inactive	Inactive
	Neurotoxicity	Active	Active	Inactive
	Immunotoxicity	Inactive	Inactive	Inactive
	Nephrotoxicity	Inactive	Inactive	Active
	Respiratory toxicity	Active	Active	Active
	Mutagenicity	Active	Inactive	Inactive
	Cardiotoxicity	Inactive	Inactive	Inactive
	Carcinogenicity	Inactive	Inactive	Inactive
	Clinical toxicity	Inactive	Active	Inactive
	Cytotoxicity	Inactive	Inactive	Inactive
	Ecotoxicity	Inactive	Active	Active
	Nutritional toxicity	Inactive	Inactive	Active
	Toxicity class	5	3	5
	AMES toxicity	Yes	No	No
	Skin sensitization	Yes	No	No
	Predicted LD_50_ (mg/kg)	24,599	200	5000
	Maximum‐tolerated dose (human) (log mg/kg/day)	0.727	0.075	0.385

### 3.2 Animal Study Findings

No mortality or severe toxicity symptoms were observed in any treatment group. All animals displayed normal physiological activity throughout the experiment, with no significant behavioral abnormalities noted.

#### 3.2.1 Marble‐Burying Capacity

Table 4 suggests that TAN significantly (*p* < 0.05) increased the number of marbles buried (NMB) as compared to the control group. TAN at the highest dose (20 mg/kg) buried 13.60 ± 3.21 marbles, whereas the control buried 9.80 ± 2.39 marbles. The dopaminergic agonist drug DOP‐22 also significantly (*p* < 0.05) increased NMB (13.00 ± 3.46), whereas the dopaminergic antagonist drug OLN‐2 reduced NMB value (9.20 ± 2.28) in comparison to the control group. TAN‐10, when co‐treated with DOP‐22, increased the NMB (14.60 ± 2.41), whereas it decreased the NMB value with the OLN‐2 (5.80 ± 2.39) group. The reference combination group (DOP‐22 + OLN‐2) showed an NBM value of 10.00 ± 2.24, which was in between the OLN‐2 and DOP‐22 groups.

**TABLE 4:** Immobility time observed in test samples and control groups.

Treatment groups	NMB	ADR (g)	TRP (s)
Control	9.80 ± 2.39	115.40 ± 5.41	10.60 ± 2.07
DOP‐22	13.00 ± 3.46*^bc^	144.00 ± 9.06*^bc^	7.00 ± 2.55*^bc^
OLN‐2	9.20 ± 2.28^c^	42.40 ± 5.03	14.60 ± 4.45
TAN‐10	7.00 ± 3.08	64.80 ± 7.79^b^	9.40 ± 1.14*^b^
TAN‐20	13.60 ± 3.21*^abc^	212.40 ± 9.21*^abc^	5.40 ± 3.05*^abc^
DOP‐22 + OLN‐2	10.00 ± 2.24*^bc^	81.20 ± 9.65^bc^	8.80 ± 3.03*^bc^
TAN‐10 + DOP‐22	14.60 ± 2.41*^abc^	153.80 ± 10.28*^abc^	6.00 ± 1.58*^abc^
TAN‐10 + OLN‐2	5.80 ± 2.39	30.40 ± 3.05	17.20 ± 3.03

*Note*: Values are mean ± SD (*n* = 5); one‐way ANOVA followed by *t*‐students Newman–Keuls as a post hoc test with multiple comparison; *p* *< *0.05 compared to the *control, ^a^DOP‐22, ^b^OLN‐2, and ^c^TAN‐10 group; control: vehicle (distilled water containing 0.9% NaCl and 0.5% Tween 80).

Abbreviations: ADR, amount of dust removed; DOP, dopamine; NMB, number of marbles buried; OLN, olanzapine; TAN, tangeretin; TRP, time to reach the target point.

#### 3.2.2 Dust Removal

In the dust removal study, the control group animals showed an ADR value of 115.40 ± 5.41 g. TAN significantly (*p* < 0.05) increased ADR values, where at 20 mg/kg, it showed the highest ADR (212.40 ± 9.21 g), which was also the highest ADR value among all the test groups. DOP‐22 also significantly (*p* < 0.05) increased ADR (144.00 ± 9.06), whereas OLN‐2 reduced the ADR value (42.40 ± 5.03) in comparison to the control group. TAN‐10, when co‐treated with DOP‐22, increased the ADR (153.80 ± 10.28), whereas it decreased the ADR value with OLN‐2 (30.40 ± 3.05). The reference combination group (DOP‐22 + OLN‐2) showed an ADR value of 81.20 ± 9.65, which was in between the OLN‐2 and DOP‐22 groups (Table 4).

#### 3.2.3 Trained Swimming Effect

Findings of the trained‐swimming study suggest that control group animals took 10.60 ± 2.07 s to reach TRP. TAN significantly (*p* < 0.05) decreased TRP values, where at 20 mg/kg, it showed the lowest TRP (5.40 ± 3.05 s), which was also the lowest TRP value among all test groups. DOP‐22 also significantly (*p* < 0.05) decreased TRP (7.00 ± 2.55 s), whereas OLN‐2 augmented it (14.60 ± 4.45 s) in comparison to the control group. TAN‐10, when co‐treated with DOP‐22, decreased the TRP (6.00 ± 1.58 s), whereas with OLN‐2 it increased the TRP value at the highest level (17.20 ± 3.03 s). The reference combination group (DOP‐22 + OLN‐2) showed a TRP value of 8.80 ± 3.03 s, which was in between the OLN‐2 and DOP‐22 groups (Table 4).

**FIGURE 4:**
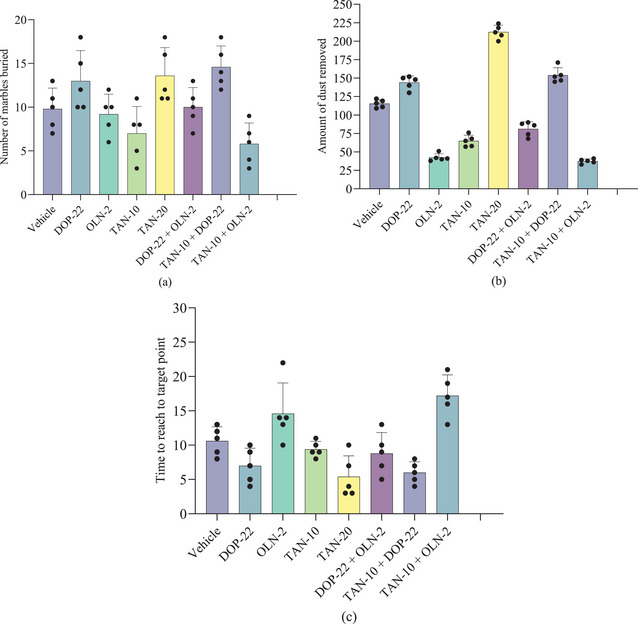
(a) Number of marbles buried; (b) amount of dust removed; (c) time to reach to the target point observed in different treatment groups of animals.

However, Figure 4 also presents the marble‐burying capacity, the dust removal study results, and the trained swimming effect study results.

## 4 Discussion

Many modulation mechanisms have been explored for memory enhancement. Neuromodulators, which change cellular and synaptic characteristics via broad projections, are well recognized to have a role in memory function (Kupfermann 1979; Floresco and Jentsch 2011). DOP is a well‐known neuromodulator that affects cognition. There is a significant increase in DOP activity in patients with disorders that affect memory (Stern and Alberini 2013). Therefore, it is generally accepted that DOP plays a critical role in working memory tasks that involve the prefrontal cortex (PFC). The capacity to record sensory inputs, events, information, etc., store it for a short or long time, and retrieve it later when needed is known as memory. In today's demanding and competitive environment, poor memory, poor retention, and delayed recall are frequent issues (Vasudevan and Parle 2007). Memory loss, cognitive impairment, anxiety, high blood pressure, dementia, and more serious disorders like schizophrenia and AZD can all be caused by age, stress, and emotions (Mani and Parle 2009).

The marble‐burying test is commonly used to assess anxiety‐related behaviors and compulsive tendencies, which are indirectly linked to memory (De Brouwer et al. 2019). Reductions in marble‐burying activities are associated with lower levels of anxiety and can enhance memory. In this investigation, the number of marbles buried rose when TAN was used, with the highest dose exhibiting the most effect. DOP‐22 also enhanced marble burial, whereas the antagonist OLN‐2 did not change. Marble burying increased when TAN and DOP‐22 were combined, indicating a synergistic dopaminergic action. However, the marble‐burying capability was decreased when TAN and OLN‐2 were coupled, indicating the inhibitory effect of OLN‐2. Correspondingly, the dust removal test involves having an animal remove dust from its fur to assess the animal's cognitive function, attention span, and memory (Moghaddam and Krystal 2012). Grooming is a complicated task that involves coordination and remembering of routine behaviors; therefore, an animal's quicker start to grooming or more thorough cleaning suggests enhanced memory and cognitive function. Higher dosages of TAN demonstrated stronger effects in the dust removal test, considerably enhancing the dust removal capacity. OLN‐2 played an adversarial function in dust clearance, but DOP‐22 enhanced it. High dust removal capability was maintained when TAN and DOP‐22 were combined, but this impact was reduced when OLN‐2 was added. When dopaminergic activation and antagonistic interactions interact, the reference group exhibits intermediate results.

**FIGURE 5:**
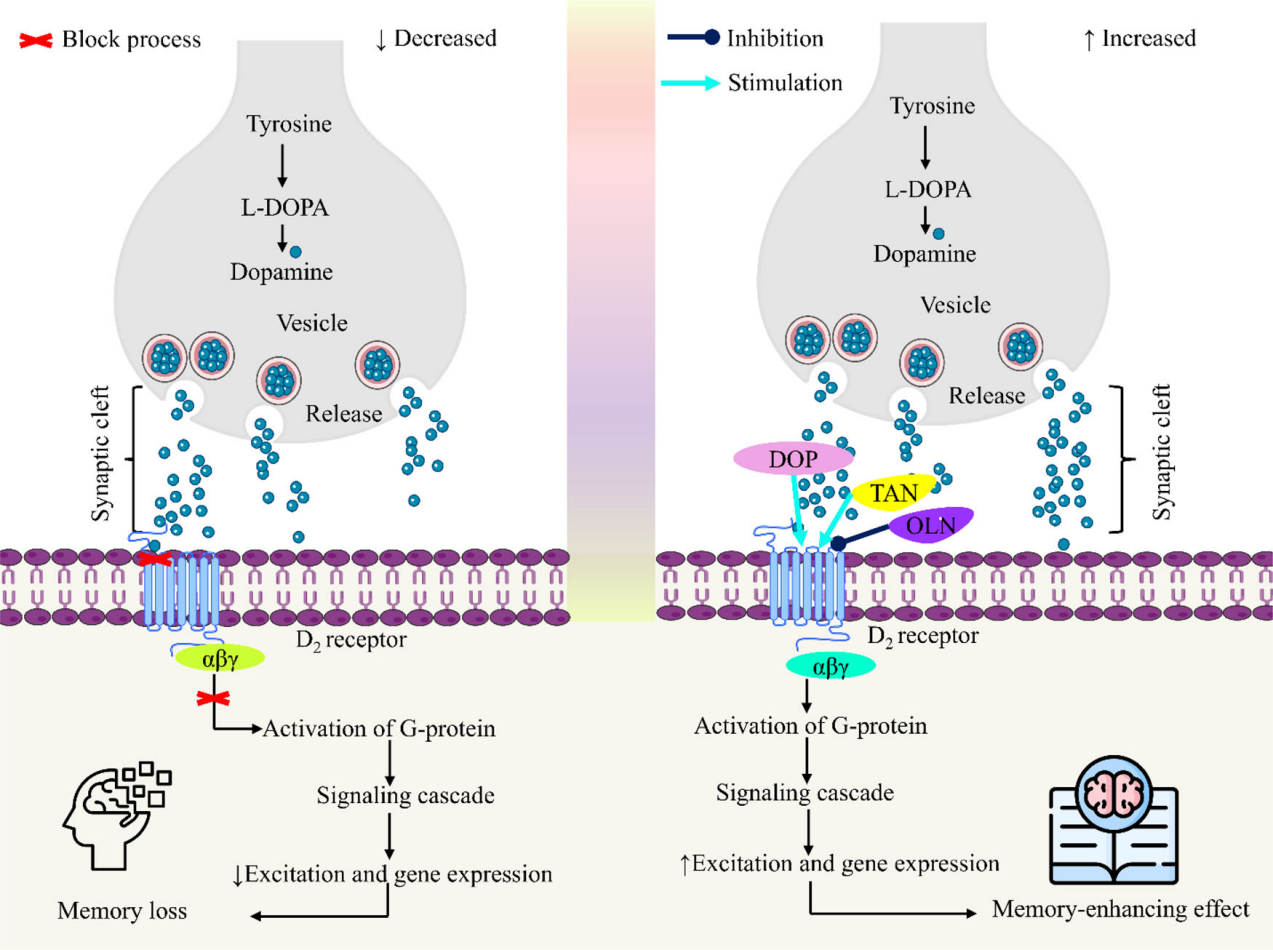
The possible memory‐enhancing effect mechanism of tangeretin. TAN alone involves its interaction with the D_2_ receptor, enhancing dopamine signaling by promoting G‐protein activation, leading to increased excitation and gene expression, which supports memory function. When TAN is combined with DOP, the effect is amplified as DOP further boosts dopamine availability in the synaptic cleft, enhancing D₂ receptor activation. This synergistic interaction strengthens the signaling cascade, resulting in greater neuronal excitation and gene expression, ultimately leading to a more pronounced memory‐enhancing effect. DOP, Dopamine; OLN, Olanzapine; TAN, Tangeretin.

A common assessment tool for learning and spatial memory is the trained swimming test, such as the Morris Water Maze (Othman et al. 2022). Multiple trials are used to record the latency, or the amount of time it takes to find the platform; faster times signify better learning. The platform is taken out of the environment, and the amount of time spent there is recorded to evaluate memory retention. Improved behavioral performance and a decrease in anxiety‐related reactions demonstrated improved learning and memory through the evaluation of anxiety reduction, cognitive enhancement, and motor coordination (Oroszi et al. 2022). In the trained swimming test, TAN enhanced swimming ability and decreased the time required to reach the target. Performance was similarly improved by DOP‐22 and decreased by OLN‐2. Although co‐treatment with OLN‐2 produced the worst performance, suggesting OLN‐2's antagonistic effect on the dopaminergic system, the combination of TAN and DOP‐22 led to additional improvement. Additionally, one study reported that TAN at 50, 100, or 200 mg/kg oral doses for 3 days showed a protective effect against 1‐methyl‐4‐phenyl‐1,2,3,6‐tetrahydropyridine (MPTP)‐induced PD in male Sprague–Dawley rats through reducing dopaminergic degeneration and hippocampal neuronal loss. It also reduced expression of inflammatory mediators’ cyclooxygenase (COX)‐2, inducible nitric oxide synthase (iNOS), as well as interleukin (IL)‐1β, 6, and 2, suggesting TAN might be a hopeful candidate to prevent neuroinflammation and dementia associated with PD (Yang et al. 2017). These findings also suggest that TAN can be a neurological agent. Moreover, the findings indicate that TAN had powerful dopaminergic effects in all tests, which were amplified by the agonist DOP‐22 and prevented by OLN‐2. These results underscore the complicated nature of TAN's interactions with other medications while indicating that it could be helpful for disorders requiring dopaminergic activity. However, Figure 5 illustrates the possible memory‐enhancing effect mechanism of TAN.

The process of finding and developing a novel medicine is well‐recognized as being extremely difficult and requiring a significant investment of time and money (Surabhi and Singh 2018). Thus, computer‐aided drug design techniques are being employed extensively to improve the effectiveness of the drug discovery and development process (Hassan Baig et al. 2016). This method reduces the requirement for laboratory animals and related expenses in addition to shortening the total amount of time needed for evaluation (Meng et al. 2011). The degree of interaction may be determined by measuring the BA between a ligand and a receptor (Azam and Abbasi 2013). The results of the docking investigation demonstrated that OLN had the strongest BA (−7.5 kcal/mol) for the target receptor, followed by TAN with a BA of −6.6 kcal/mol. On the other hand, the standard compound DOP exhibited a lower BA of −5.0 kcal/mol. The HB interactions are essential for the formation of stable ligand–receptor complexes, significantly influencing ligand selectivity (Xu et al. 2014). Moreover, HB enhances ligand binding strength and therapeutic efficacy (Lu et al. 2012). In this study, DOP formed three HBs with ASN A: 430, ASN A: 70, and GLU A: 368, contributing to its interaction stability. However, despite the higher number of HBs, DOP's BA was weaker compared to TAN and OLN. TAN formed one HB with ALA A: 379, but its stronger BA is likely due to additional hydrophobic interactions with residues like ILE A: 383 and PHE A: 202, which provided greater stability to the ligand–receptor complex. TAN also shared similar non‐bonding interactions with GLU A: 368, as seen in DOP, suggesting a common binding motif but with enhanced efficacy. OLN, with its single HB to TYR A: 209 and multiple hydrophobic interactions, exhibited the highest BA, indicating its superior binding strength and potential as a therapeutic agent. The combined results highlight the critical role of both HBs and hydrophobic interactions in determining the binding efficiency of these ligands, with TAN and OLN outperforming DOP in terms of receptor binding and stability.

Pharmacokinetics is the scientific term for the study of the temporal dynamics related to drug disposition in the human body, which includes drug molecule absorption, distribution, metabolism, and elimination processes (Chowdhury et al. 2024; Al Hasan et al. 2025). It assesses the physicochemical characteristics of the substance to ascertain its pharmacokinetic concerning nature (Camp et al. 2015). A popular method for forecasting pharmacokinetics and drug‐likeness is Lipinski's rule of five. A drug candidate should meet the criteria of Lipinski's rule of five, which is as follows: MW of 500 g/mol or less; HBA and HBD values of TAN and DOP within the limitations (HBA < 10, HBD ≤ 5); and lipophilicity (Log P_o/w_) of no more than five (Rani et al. 2023). Furthermore, the MR of TAN and DOP, the two ligands, is within the range (MR ≤ 140) at 100.38 and 42.97, respectively. By using Lipinski's criterion, all ligands are predicted to have outstanding pharmacokinetic qualities and to be in the range of becoming possible medicines. The selected test ligand exhibits improved pharmacokinetic qualities and satisfies every requirement listed in Lipinski's rule of five. In silico toxicity studies are crucial for the creation of safe and cost‐effective pharmaceuticals (Marques et al. 2024). Furthermore, it was essential to identify any potential negative effects that substances could have through toxicology testing (Krewski et al. 2020). The results of the analysis show that TAN has no negative impacts on immunotoxicity, hepatotoxicity, carcinogenicity, cardiotoxicity, neurotoxicity, or clinical impacts. However, it did show negative side effects on several organs such as nephrotoxicity, respiratory toxicity, ecotoxicity, and nutritional toxicity.

Combination therapy involves using two or more treatments together to enhance effectiveness and reduce resistance (Khan 2019). This approach is important in modern medicine as it allows for a more comprehensive strategy, improving overall outcomes and minimizing side effects by targeting different mechanisms simultaneously (Mokhtari et al. 2017). Combination therapy with TAN and the dopaminergic agonist DOP‐22 significantly (*p* < 0.05) improves behavioral outcomes, increasing the NMB and ADR while reducing the TRP. The co‐treatment shows a synergistic effect, enhancing therapeutic benefits compared to control and monotherapy. This underscores the value of combination strategies in optimizing treatment outcomes.

Overall, in vivo studies demonstrated that TAN significantly (*p* < 0.05) enhanced behaviors such as marble‐burying capacity, dust removal, and reduced target reach time, highlighting its efficacy in combination therapy settings. Moreover, in silico analyses revealed that TAN and OLN exhibited a stronger BA for the D_2_ receptor, outperforming the binding affinities of DOP. Additionally, TAN displayed favorable pharmacokinetic properties, including good absorption and lower toxicity profiles compared to both DOP and OLN. These findings collectively underscore TAN's potential as a promising therapeutic agent, suggesting it may be advantageous in future clinical applications.

## 5 Conclusion

In conclusion, the present study provides strong evidence supporting the memory‐enhancing effects of TAN in both in vivo and in silico models. TAN significantly (*p* < 0.05) improved cognitive functions in *Swiss* albino mice, enhancing memory, anxiety, and motor coordination. TAN‐20 mg/kg led to notable improvements, including increased NMB (13.60 ± 3.21), enhanced ADR (212.40 ± 9.21 g), and TRP (5.40 ± 3.05 s). The combination of TAN‐10 mg/kg with DOP‐22 mg/kg further boosted cognitive performance, suggesting a synergistic effect. In silico docking studies revealed a strong BA of TAN (−6.6 kcal/mol) for the D_2_ DOP receptor, forming three HBs and several non‐covalent interactions, suggesting that its neuroprotective and cognitive benefits may be attributed to dopaminergic modulation. Additionally, TAN exhibited a favorable pharmacokinetic profile with high absorption, bioavailability, effective BBB penetration, and compliance with Lipinski's Rule of Five, which enhances its potential as a therapeutic agent. In toxicity assessments, TAN demonstrated low toxicity, with an LD_50_ value of 5000 mg/kg and no significant hepatotoxicity or carcinogenicity risks. This study is limited by the short experimental duration and the lack of human clinical trials. The focus on the dopaminergic system excludes other neurotransmitter pathways, and in vivo toxicity assessments were not conducted. Additionally, species‐specific differences may limit the direct translation of findings to humans. However, findings of this study offer a promising outlook for the use of TAN in the treatment of memory‐related disorders, warranting further clinical studies to explore its full therapeutic potential in neurodegenerative diseases.

## Author Contributions

**Md. Sakib Al Hasan**: conceptualization, investigation, methodology, writing–original draft, writing–review and editing, formal analysis. **Mohd Shahnawaz Khan**: conceptualization, writing–review and editing, funding. **Arusha Ayub**: conceptualization, writing–review and editing, writing–original draft. **Raihan Chowdhury**: conceptualization, validation, methodology. **Emon Mia**: methodology, investigation, writing–original draft, writing–review and editing, formal analysis. **Md. Shadin**: software, visualization, resources. **Md. Showkot Akbor**: data curation, resources, visualization. **Muhammad Torequl Islam**: conceptualization, investigation, writing–original draft, writing–review and editing, visualization, validation, methodology, software, formal analysis, project administration, resources, supervision, data curation. **Md. Shimul Bhuia**: writing–original draft, conceptualization, writing–review and editing.

## Ethics Statement

This study was approved by the Animal Ethics Committee of Khulna University (KUAEC‐2023‐05‐09).

## Consent

The authors have nothing to report.

## Conflicts of Interest

The authors declare no conflicts of interest.

### Peer Review

The peer review history for this article is available at https://publons.com/publon/10.1002/brb3.70516

## Permission to Reproduce Material From Other Sources

The authors have nothing to report.

## Data Availability

Data will be made available on request.
